# First case of new-onset psychosis due to DBS in the ventralis intermediate nucleus (Vim) of the thalamus

**DOI:** 10.1192/j.eurpsy.2022.643

**Published:** 2022-09-01

**Authors:** S. Petrykiv, M. Arts, L. De Jonge

**Affiliations:** 1 GGZWNB, Psychiatry, Halsteren, Netherlands; 2 GGZWNB, Psychiatry, Bergen op Zoom, Netherlands; 3 Leonardo Scientific Research Institute, Neuropsychiatry, Bergen op Zoom, Netherlands

**Keywords:** Psychosis, Treatment, DBS, Therapy resistance

## Abstract

**Introduction:**

Known risk factors for developing of first-time psychosis in patients with deep brain stimulator (DBS) include older age, short time after implant placement and cerebral target for stimulation. In particular, stimulation of subtalamic nucleus and globus pallidus internus has been shown to elicit psychotic symptoms in various case reports. To date, there are no cases describing onset of psychosis due to DBS in the ventralis intermediate nucleus (Vim) of the thalamus.

**Objectives:**

Case describtion of psychotic episode provoked by DBS in Vim region

**Methods:**

Case report of 70 y.o. female with unilateral DBS from 2012 in Vim for essential tremor, who developed therapy resistant psychotic symptoms right after adjusted settings of the DBS.

**Results:**

Psychotic onset of otherwise healthy 70 y.o. patient occurred and gradually worsened after adjustment of DBS settings in absence of other iatrogenic factors, including medication and comorbidity, and required involuntary hospitalization one week after beginning of psychosis. Treatment after hospitalization comprised olanzapine 10 mg. 1dd1 did not cause resolvent of psychosis. Because of therapy resistance to psychofarmaca and worsening of psychotic symptoms, by way of exception neurologists had to change the settings back to basic leading to complete and sustained remission of psychosis within two days.

**Conclusions:**

Among side effects of DBS in Vim, psychotic symptoms have never been reported. However, as in our patient, psychosis occurred after changes of settings in DBS and presented acutely, was severe, resulted in involuntary hospitalization and was therapy resistant. Pathophysiology of DBS-induced psychosis in Vim region is not known and requires further investigation.

**Disclosure:**

No significant relationships.

